# Identifying Adaptations to an mHealth Alcohol Reduction Intervention for Reducing Alcohol Use in Adolescent and Young Adult Cancer Survivors: Qualitative Study

**DOI:** 10.2196/59949

**Published:** 2025-08-07

**Authors:** Kimberly Haney, Tia Borger, Vilma Bursac, Caryn Sorge, Brent Shelton, John Salsman, Laurie McLouth, Carolyn Lauckner

**Affiliations:** 1 Center for Health Equity Transformation, University of Kentucky, Suite 460, Healthy Kentucky Research Building, 760 Press Avenue, Lexington, KY, 40508, United States, 1 859-302-2178; 2 Department of Psychology, College of Arts and Sciences, University of Kentucky, Lexington, KY, United States; 3 Department of Pediatrics, College of Medicine, University of Kentucky, Lexington, KY, United States; 4 Division of Cancer Biostatistics, Department of Internal Medicine, College of Medicine, University of Kentucky, Lexington, KY, United States; 5 Department of Social Sciences and Health Policy, School of Medicine, Wake Forest University, Winston-Salem, NC, United States

**Keywords:** alcohol use, young adults, cancer survivors, mobile health, mobile phone, mHealth, motivational interviewing, intervention, alcohol reduction, alcohol, adolescent, adult, young adult, cancer, mental health, physical health, tracking, treatment, oncology, psycho-oncology

## Abstract

**Background:**

Adolescent and young adult survivors of cancer (aged 15‐39 years) frequently engage in hazardous alcohol use, which can have multiple mental and physical health effects.

**Objective:**

The aim of this study was 2-fold, to identify the necessary adaptations to an existing motivational interviewing-based mHealth (mobile health) alcohol reduction intervention, called Tracking and Reducing Alcohol Consumption (TRAC), for posttreatment adolescents and young adults, and to develop a tailored intervention for this vulnerable and underserved population.

**Methods:**

This was a qualitative study consisting of key informant interviews with posttreatment adolescents and young adults aged 18‐39 years, oncology and psych-oncology providers, and community advocates (n=15) to inform the adaptation of TRAC. Thematic qualitative analysis of interview findings was conducted to determine necessary changes to the intervention protocol and content, which would ultimately lead to the development of the new TRAC adolescent and young adult intervention.

**Results:**

Key informant interviews revealed a need for the intervention to address cancer-specific alcohol use triggers such as scan-related anxiety, financial toxicity, and reproductive health concerns. They also indicated the need to provide education on the link between alcohol and cancer and to reduce the time burden of the intervention, given the many competing life demands of adolescents and young adults. Significant changes were made to the TRAC intervention to create the TRAC adolescent and young adult. We reduced the number of required sessions from 8 to 4, introduced a session devoted to managing cancer-specific triggers, and provided survivors with more information about alcohol and its relationship to cancer.

**Conclusions:**

There is potential to increase alcohol intervention relevance and fit for adolescents and young adults by including tailored content relevant to their life experiences while also maintaining core components of such interventions, such as self-monitoring and goal-setting. Remote, brief interventions are important for ensuring acceptability. The new TRAC adolescent and young adult intervention represents a potentially valuable tool in addressing high rates of hazardous alcohol use among this population and warrants further evaluation in randomized trials.

## Introduction

### Background

Each year in the United States, nearly 90,000 people aged 15‐39 years (adolescents and young adults) are diagnosed with cancer [1]. A total of 5-year survival for adolescents and young adults is over 80%; most will live an additional 36‐59 years after diagnosis [1], highlighting the critical importance of maximizing their physical and mental health to support quality of life and avoid premature mortality. Unfortunately, adolescent and young adult cancer survivors report poorer physical and mental health compared with their unaffected peers and have a greater than 2-fold risk of dying from noncancer-related causes, such as cardiovascular disease [2-5].

Hazardous alcohol use (ie, heavy or binge drinking) is one modifiable factor that may contribute to adolescent and young adult cancer survivors’ physical and mental health. Over 50% of adolescent and young adult cancer survivors exceed moderate drinking recommendations [6], and 36%‐45% of survivors report engaging in binge drinking, defined as consuming 5 or more drinks (for males) or 4 or more drinks (for females) in a 2-hour period [6,7]. Given known health risks of hazardous alcohol use in the general population (eg, increased risk of heart and liver disease, stroke, depression and anxiety, and reduced fertility), as well as in cancer survivors specifically (eg, increased risk of certain cancer incidence, recurrence, and even premature mortality) [2-5], helping adolescent and young adult cancer survivors reduce hazardous drinking should be a key component of survivorship care.

Unfortunately, few interventions have been developed and tested to address risky alcohol use among cancer survivors, let alone among adolescent and young adult cancer survivors. Evidence of the effectiveness of such interventions is limited, with many studies focusing on general behavioral health interventions rather than alcohol use specifically [8]. Few have leveraged mHealth (mobile health) or similar technology-based interventions that may significantly increase reach not only among adolescent and young adult cancer survivors who show near ubiquitous use of smartphones [9], but also among those living in rural and underserved areas who have limited access to mental health providers [10] and may experience stigma when seeking help [11,12]. Finally, no interventions have been developed to address the potentially distinct needs of adolescent and young adult cancer survivors who, by virtue of their developmental and life stage, may face additional social pressure to drink and may experience unique stressors that may trigger drinking, such as mental health concerns stemming from their cancer experience [13].

### Objectives

The purpose of this study was to identify modifications needed for a previously developed mHealth intervention, originally designed to reduce alcohol use among people living with HIV (“TRAC”) and shown to have high acceptability, feasibility, and significant pre-post effects on alcohol use in a pilot trial [14]. We also sought to develop a new, tailored intervention for posttreatment adolescent and young adult cancer survivors. We focused on the posttreatment period to ensure survivors had experienced sufficient time to establish more routine drinking patterns, as well as to avoid the likelihood that active cancer treatment side effects may impact drinking levels [15,16]. In addition, we specifically examined rural adolescent and young adult cancer survivor experiences and needs to ensure the resulting intervention would reach this underserved, vulnerable population.

## Methods

### Framework and Study Design

This study was part of a larger project aimed at developing an mHealth intervention to reduce hazardous alcohol use among posttreatment adolescents and young adults that was approved by the University of Kentucky Institutional Review Board (67410). We used Escoffery et al’s [17] synthesis of the common steps outlined across adaptation frameworks to guide overall study design. The formative qualitative research described in this paper was guided by the following adaptation steps: understand the needs of the new target population (posttreatment adolescents and young adults) with respect to alcohol interventions, determine how core components of the original intervention aligned with the new population context, consult with subject experts and stakeholders, and determine potential changes in the original intervention’s structure, content, and delivery methods to ensure alignment between the intervention and needs of adolescents and young adults. Steps of pretesting and pilot testing the adapted intervention were achieved through an open pilot and pilot randomized controlled trial, which will be described in a future paper. The COREQ (Consolidated criteria for Reporting Qualitative research) checklist [18] was used to guide reporting throughout this manuscript and is included as Checklist 1.

### Description of Existing Intervention (Tracking and Reducing Alcohol Consumption)

The original Tracking and Reducing Alcohol Consumption (TRAC) intervention was designed as an alcohol reduction intervention for individuals living with HIV. It focused on increasing motivation to reduce hazardous drinking and awareness of drinking behaviors, building skills to reduce alcohol use, and identifying general triggers for drinking. In this original intervention, there was minimal discussion surrounding potential HIV-specific triggers for drinking [14]. Over an 8-week period, 8, 30-minute sessions were conducted remotely via video chat or telephone call with a trained interventionist (Can be found in Table 1 for session content of the original TRAC intervention). Throughout the intervention period, participants completed twice-daily self-monitoring of alcohol use via mobile surveys and a BACtrack Mobile Breathalyzer, which uses law enforcement-grade sensors to determine breath alcohol concentration (BrAC). The study interventionist discussed the previous weeks’ alcohol use as reported via these self-monitoring tasks during weekly intervention sessions.

**Table 1.** Original Tracking and Reducing Alcohol Consumption intervention (preinterviews).

Session number	Session emphasis	Session content
Session 1	Developing a change plan	Review current drinking behaviors, when or why they drink Identify reasons for changing drinking behaviors (change plan) Self-monitoring and phone training
Session 2	Triggers for drinking alcohol	Urges and cravings Emotional triggers Situational and environmental triggers Social triggers
Session 3	Skill building: distraction	Breathing awareness Personal distraction techniques
Session 4	Skill building: managing urges and cravings	Delay before acting Negative consequences of drinking Positive consequences of not drinking
Session 5	Skill building: managing emotional triggers	Improve the moment Do something relaxing
Session 6	Skill building: managing social triggers	Drink refusal Seek social support Seek spiritual support
Session 7	Skill building: managing situation and environmental triggers	Consuming alternate food or drink Engaging in alternate behavior Avoiding the situation or environment
Session 8	Looking ahead	Summative self-assessment Planning for the future

### Participants

Key informant interview participants (n=15) included adolescent and young adult cancer survivors, providers, and community advocates to represent the perspectives of key stakeholders and subject experts. These partners were chosen because they were expected to offer important perspectives on the unique psychosocial needs adolescents and young adults might face that could influence drinking, the health care needs pertaining to psychosocial interventions to address alcohol use among adolescents and young adults, and the specific challenges adolescents and young adults from rural and underserved areas might experience that could influence intervention acceptability and feasibility. Oncology and psycho-oncology providers were selected to determine what clinical resources for discussing alcohol use are used with their patients, if any, and to hear provider perspectives on potential intervention content. Community advocates and survivors were selected based on their work with or experience as adolescent and young adult survivors of cancer and their insight regarding unmet needs of this population.

Recruitment was accomplished via convenience sampling. We used the University of Kentucky Markey Cancer Center Community Impact Office to identify community advocates, professional networks to identify oncology and psycho-oncology providers, and reviewed clinic schedules at the University of Kentucky Markey Cancer Center to identify potentially eligible survivors. We also contacted survivors who recently completed a related survey and had indicated interest in participating in follow-up research. Survivors were eligible if they were between the ages of 18 and 39 years, had been diagnosed with cancer between the ages of 15 and 39 years and identified as a survivor of cancer (ie, had completed cancer treatment), and were residents of a rural area (determined by the zip code of their primary residence). Oncology providers included physicians, nurses, nurse practitioners, or physician assistants who had been in practice for at least 6 months and saw at least 25% adolescent and young adult cancer patients in their practice. Psycho-oncology providers included social workers, psychologists, or other mental health providers who treated adolescent and young adult cancer patients and had been in practice for at least 6 months. Community advocates included partners from the Kentucky Cancer Consortium and the Kentucky American Cancer Society who had previous experience with adolescent and young adult populations and the specific challenges that come with living in rural Kentucky. Individuals who were unable to speak English were excluded from the key informant interviews. Participants were approached either in person or remotely through email or phone calls and completed a brief screening survey to determine eligibility. All participants provided documentation of informed consent via REDCap (Research Electronic Data Capture) [19,20] before beginning the interview. Key informants received US$40 as compensation for their participation.

#### Sample Size Considerations

Sample size is often driven by factors beyond saturation [17], and in this case, the sample size was driven by the goal of the interviews, which was to include different partner perspectives on the development of a new, tailored intervention. Other considerations for sample size for the interviews included pragmatic concerns related to time and resource constraints in the context of the larger 2-year project, as these interviews were but the first phase in a series of studies designed to develop and pilot this new intervention. After completion of the interviews, we conducted a small open pilot to refine protocols (n=4), followed by a randomized pilot trial (N=40) that sought to evaluate feasibility and acceptability of intervention content; analysis of the full results of these remaining phases is ongoing. Thus, the short timeline, limited resources, and the goals of the research (ie, to hear different perspectives on the new intervention) guided decisions regarding overall sample size and recruitment efforts for the interviews. The implications of this approach are discussed in more detail in the Limitations section.

### Interview Protocol

Key informant interviews lasted between 45 and 60 minutes and were conducted through videoconferencing, by phone, or in-person at a clinic using a semistructured interview guide developed by the principal investigators. In-person interviews were completed in a private room with only the interviewer and participant present. Participants who completed the interview remotely (either via phone call or videoconference) were instructed to find a private location for the interview. All interviews were audio-recorded and transcribed verbatim, supplemented by field notes made by the interviewer during the interview. Participants did not receive copies of these transcripts or audio files. There were no follow-up interviews or surveys. The semistructured interview guides, which contained over 20 questions with follow-up probes, elicited feedback on the relevance, appropriateness, acceptability, and feasibility of TRAC content and delivery. Refer to Table 2 for highlighted interview questions that yielded some of the more informative responses. The interviewer was a male research staff member trained on the interview protocol by the principal investigators, who had a Master’s degree in Social Work and experience working in substance use treatment settings. He provided reference materials to the participants before the interview, including a diagram of the study steps and components and excerpts from the participant manual. Individuals from all 3 participant groups were generally asked the same questions, though there were some differences. Oncology and psycho-oncology providers were asked questions about their approaches to assessing and counseling on alcohol use in their practice. Survivors were asked whether they had received any information or recommendations from their providers about maintaining a healthy lifestyle posttreatment, what those recommendations were, and if they had been told about alcohol’s relationship to cancer recurrence or development of other cancers. In addition to the interview, key informants also completed a brief demographic questionnaire that collected information on gender, race and ethnicity, and education and income level.

**Table 2.** Key informant interview domains.

Interview domain	Participant group	Example interview questions	Relevant adaptation step^a^		
			a	b	c
Content and Components	All groups	Overall concept of intervention for adolescents and young adults: Do you think that adolescent and young adult cancer survivors would be interested in participating in a program like this?	✓	✓	✓
		Adding information on alcohol and cancer survivorship for motivation to change: How helpful do you think the information is about the relationship between alcohol and cancer, if at all?	✓		✓
		Unique stressors adolescents and young adults face that are relevant to alcohol urges and cravings, drink refusal skills: Are there unique stressors or circumstances faced by this population that could impact drinking?	✓		✓
		Breathing and stress reduction techniques: Do you think the breathing exercise is a useful skill to cover in the first session?			✓
		Use of breathalyzers, frequency: How do you think survivors will feel about using mobile breathalyzers to track alcohol use?	✓	✓	✓
	Survivors	Recommendations from providers after finishing cancer treatment: What recommendations, if any, have providers given you about staying healthy postcancer treatment?	✓		
		Discussion with providers about alcohol’s relationship to cancer: What sort of information, if any, have you received about alcohol use and its relationship to cancer recurrence?	✓	✓	✓
	Oncology providers	Recommendations to patients about alcohol use after finishing cancer treatment: What recommendations, if any, do you give to patients about alcohol use posttreatment?	✓		
		Information patients receive about alcohol use and cancer: What sort of information, if any, do patients routinely receive about alcohol use and the risk of cancer recurrence or second malignancies?	✓	✓	✓
	Psycho-oncology Providers	Discussion of alcohol use with patients after finishing cancer treatment: How often do you discuss alcohol use with posttreatment adolescent and young adult cancer survivors? What recommendations, if any, do you provide to survivors regarding alcohol use?	✓	✓	✓
Delivery	All groups	Number of sessions, session length: What do you think about this 8-session schedule?	✓		✓
		Pros and cons of using smartphones: What do you see as the pros and cons of using phones for delivery?	✓		✓
		Strategies for retention, adherence: What ideas do you have for helping to keep survivors engaged in the program and attending sessions?	✓		✓
	Survivors	Interventionist characteristics: Who do you think would be the best type of person to deliver these counseling sessions?	✓		✓

a Adaptation Steps: (a) Understand needs of posttreatment adolescents and young adults with respect to alcohol interventions; (b) determine core component alignment; (c) determine potential changes in structure, content, and delivery methods

### Analysis

Interview transcripts were analyzed using a thematic approach put forth by Braun and Clarke [21]. Analysis steps involved: (1) reading and discussing two randomly selected interviews, (2) identifying key concepts as preliminary codes, (3) creating a draft codebook with operational definitions for each code, (4) double-coding the initial two randomly selected interviews by line-by-line coding (ie, independent coding by TB and KH, with discrepancies rectified to achieve a final set of agreed-upon codes for each interview), (5) finalizing the codebook, (6) revising the initial two previously coded interviews to reflect the final codebook, (7) double-coding all remaining interviews until we reached ≥80% agreement (4/7 survivor interviews and 5/8 provider and advocate interviews were double coded), and (8) analyzing all coded interviews in NVivo (Lumivero) [22]. In total, 2 separate codebooks were created (survivor or provider and advocate). Participants were not contacted to provide feedback on the codebooks or findings.

### Ethical Considerations

All procedures were approved by the University of Kentucky Institutional Review Board (67410). All participant data were held in confidence and kept secure, and only the approved study staff involved with the research and those responsible for overseeing the research had access to information that may identify the participants. All participants completed informed consent before beginning any research activities. Consenting was completed electronically using the REDCap eConsent framework. Participants were first given basic information about the study, and if they expressed interest, they were given the opportunity to complete a brief screening survey to determine eligibility. If participants met eligibility criteria, a member of the study team obtained their informed consent. Participants were told that they may stop their participation in the study at any time, without consequence. The research team also provided contact information for the principal investigators and the University of Kentucky’s Office of Research Integrity. Participants were encouraged to contact these individuals if they had questions or concerns regarding this research or questions about their rights as research participants.

To protect confidentiality, participants were assigned a unique study ID number. Any identifying information was not stored alongside their survey or interview responses. Electronic data were stored within REDCap. Audio files and transcripts of the interviews were stored in a password-protected HIPAA (Health Insurance Portability and Accountability Act)-compliant Microsoft OneDrive folder housed at the University of Kentucky. Only approved study staff had access to the study data. As part of this study, participants could earn up to US$40. Upon completion of the interview, participants were paid using gift cards.

## Results

### Participants

Participants included 7 adolescent and young adult cancer survivors (mean age 24 [4.8] yrs; range=19‐38; 71% female; 100% white), 3 oncologists (Mean years in practice 16 [3.5] yrs; 67% female; 67% white), 3 psycho-oncology providers, all of whom were licensed mental health practitioners (Mean y in practice 17 [3.5] yrs; 100% female, 100% white), and 2 community advocates (Mean age 45 [12.7] yrs; 50% female; 50% white; Table 3).

**Table 3.** Key informant interview participant demographics.

Characteristic	Measure
Survivors (n=7), mean (SD), years	
Current age	24 (4.8); range=19-38
Age at diagnosis	26 (4.8); range=17‐36
Gender, n (%)	
Women	5 (71)
Men	2 (29)
Race, n (%)	
White	7 (100)
Oncology providers (n=3)	
Years in practice, mean (SD)	16 (3.5)
Gender, n (%)	
Women	2 (67)
Men	1 (33)
Race, n (%)	
White	2 (67)
Unknown	1 (33)
Psycho-oncology providers (n=3)	
Years in practice, mean (SD)	17 (3.5)
Gender, n (%)	
Women	3 (100)
Race, n (%)	
White	3 (100)
Community advocates (n=2)	
Age (years), mean (SD)	45 (12.7)
Gender, n (%)	
Women	1 (50)
Unknown	1 (50)
Race, n (%)	
White	1 (50)
Unknown	1 (50)

While data on alcohol use from one survivor participant was missing, among the remaining 6 participants, 83% were current drinkers, and 67% reported past month hazardous drinking (ie, reported getting “drunk or intoxicated” at least once). Responses were similar across groups, with no major differences in perspectives between survivors, clinicians, and community advocates. The following sections describe key themes obtained from their responses.

### Theme 1: Lack of Provider Discussions About Alcohol and Need for Intervention

Survivors, oncology providers, and psycho-oncology providers all spoke about the lack of discussion about alcohol use during posttreatment and long-term follow-up care. Most oncology and psycho-oncology providers interviewed said they rarely discussed alcohol use with patients, if at all. One clinician remarked, “That’s usually something I don’t bring up,” [Oncologist, ID 14] while another said alcohol use is discussed only if it is “clinically relevant” [Psycho-Oncologist, ID 3]. Of the 6 oncology and psycho-oncology providers interviewed, only one reported discussing alcohol use with their patients, and only then to “try to educate people about the recommended dose” [Psycho-Oncologist, ID 10].

Survivors who were interviewed likewise reported that they received little to no information about alcohol use once they completed cancer treatment. “They didn’t really give me any restrictions,” said one survivor [ID 4], while another said “I have received no information about alcohol and cancer” [ID 9]. Others recalled that providers advised them to “maintain a healthy diet and exercise,” [ID 26] or “wear sunscreen,” [ID 9] for example, but did not explicitly mention alcohol.

### Theme 2: Time Commitment and Requirements of Intervention

The time commitment required of participants in the intervention was a concern for key informants, with one survivor describing the original 8-week TRAC program as “a lot for a working person” and “kind of overwhelming” [ID 68]. Other survivors expressed concerns that the time commitment may be a barrier to participation, saying “some people might think ‘I don’t want to do this for 8 weeks’” [ID 26] and “I think you’re running into people not finishing, or just not wanting to do it, because it is too time consuming” [ID 68]. Likewise, providers and community advocates also expressed concerns about the required time commitment of the intervention. “I think 8 sessions will be tough to get,” remarked one advocate [ID 18], and “doing something for an hour, you’re going to lose their interest” [Oncologist, ID 15]. Participants from all 4 groups recommended flexible approaches to the intervention to help reduce the time burden. These approaches included changing the delivery format based on personal preference, that is, allowing participants to choose 8, 30-minute sessions or 6, 40-minute sessions based on what fit best in their schedules.

Participants also expressed concern about adherence to twice-daily surveys for the full 8-week period, with one participant stating they “wonder about compliance with daily surveys, that would be one thing that might be hard for people for 8 weeks” [Psycho-Oncologist, ID 11]. Participants offered ideas to better encourage completion of these daily monitoring tasks, including monetary compensation, prize drawings, and the ability to track other health behaviors relevant to survivors.

### Theme 3: Information on Cancer Survivorship, Cancer Recurrence, and Alcohol Use

Both the oncology and psycho-oncology provider groups had strong recommendations surrounding the intervention content, specifically content about survivorship and recurrence in the context of survivors’ alcohol use. Participants from these groups suggested providing “more context about the relationship between alcohol and the cancers listed in the intervention manual” [Oncologist, ID 14]. One psycho-oncology provider suggested adding information about “specific health issues related to cancer, and how alcohol might impact those issues as well,” (eg, worsened mental health as a result of cancer diagnosis and treatment, conditions caused by drug side effects) [ID 11]. However, participants noted the need to be mindful of the wording used when discussing alcohol use and cancer to avoid triggering feelings of guilt or shame, saying “you need to make it non-judgmental right from the get go,” [Oncologist, ID 14] and “I think helping them understand how drinking can be problematic without making them feel guilty or stigmatized” [Psycho-Oncologist, ID 3].

### Theme 4: Inclusion of Cancer-Specific Triggers

All 4 groups remarked on the need to expand on the types of alcohol use triggers discussed in TRAC as there are unique circumstances and stressors which impact the lives of cancer survivors. One survivor participant stated, “There’s a potential for medical triggers—like having to go back to the doctor” [ID 4]. Another participant echoed this sentiment, saying,

There would be a benefit of including some potential triggers related to their cancer survivorship status, like, drinking to manage anxiety about follow up scans

[Psycho-Oncologist, ID 10].

Other suggestions included discussing the financial burden that many survivors face due to cancer treatment and including information relating to reproductive health concerns or fertility, with participants from multiple groups stating that these could be triggers for adolescents and young adults.

### Theme 5: Hesitation Toward Mobile Breathalyzers

The use of breathalyzers was an area of concern for interviewees, with many suggesting that intervention participants may find them off-putting. One survivor remarked, “It’s almost downgrading [sic]…they might feel bad about themselves when they do it” [ID 68], with another survivor suggesting that they would not complete a breathalyzer reading if they have been drinking because they would not want to see that recorded— “I wouldn’t be against it, but I wouldn’t actually use it. I’ve had too much to drink…do I really want that being recorded?” [ID 20]. Others expressed concerns about the data obtained from breathalyzers—whether participants would complete the readings if they had been drinking, or if participants would fabricate data to avoid feelings of judgment. One provider participant stated that “[relying] on them to be able to give you that data when they’re impaired is probably wishful thinking” [Oncologist, ID 14]. Informants recommended using neutral, nonstigmatizing language when discussing the monitoring procedures with participants. A participant from the community advocate group emphasized this, saying, “It will be important that they feel respected, valued, and safe to be willing to be that vulnerable” [ID 16].

### Adaptations Following the Key Informant Interviews

Significant changes were made to TRAC based on feedback from the key informant interviews, resulting in the new TRAC adolescent and young adult intervention. The most notable changes involved (1) reducing the length and time commitment of the intervention to accommodate the busy schedules of adolescents and young adults and (2) adding new content to address the link between alcohol and cancer and cancer-specific triggers among adolescent and young adult cancer survivors. Core components (eg, identifying and managing triggers) were maintained. Refer to Table 4 for a summary of the new intervention content and key intervention changes, discussed in more detail below.

**Table 4.** New tracking and reducing alcohol consumption in adolescents and young adults intervention.

Session number	Session emphasis	Session content	Summary of changes from TRAC^a^
Session 1	Understanding your drinking and reasons for change	Education on alcohol and cancer survivorship Review current drinking behaviors, when and why they drink Identify reasons for changing drinking behaviors (change plan) Skill: Breathing awareness, deep breathing	Removed self-monitoring and phone training (moved to orientation) Added education on alcohol and cancer survivorship Added breathing awareness and deep breathing skill training (TRAC Session 3)
Session 2	Managing your biggest trigger for drinking	Introducing concept of triggers and different types Managing emotional OR situational or social triggers Building skills and brainstorming strategies for addressing relevant triggers Skill: Riding the wave for urges and cravings Suggested activities to do instead of drinking	Combined TRAC sessions 2 and 5‐7 on emotional, situational, and social triggers into one session (participants choose which is most relevant) Added urge surfing skill building to replace TRAC Session 4 (Managing Urges & Cravings) Added list of suggested activities to do instead of drinking (TRAC Session 3)
Session 3	Understanding and managing cancer-specific triggers	Fear of recurrence and surveillance Managing financial concerns OR Reproductive health worries Building skills and brainstorming strategies for coping with relevant triggers (eg, creating a survivorship care plan, seeking support from others)	All new content developed for TRAC-adolescent and young adult
Session 4	Looking ahead	Summative self-assessment Planning for the future Finding resources in your community	Added additional resources for ongoing support

a TRAC: tracking and reducing alcohol consumption.

### Adaptations to Reduce Overall Time Commitment

TRAC (8 sessions) became TRAC adolescent and young adult (4 sessions)—collapsing the 3 sessions devoted to the types of potential triggers into one, and adding a separate session focusing solely on triggers specific to cancer survivorship. A key part of reducing the number of intervention sessions involves allowing survivors to choose which topics are most relevant to them for discussion, which also allows the intervention to feel more tailored to their needs and experiences. In the new TRAC-adolescent and young adult intervention, participants are asked to describe the context of their typical drinking experiences, and the counselor works with them to determine if they are most often drinking due to social, situational, or emotional triggers. They then focus specifically on those kinds of triggers during the intervention (versus covering all types of triggers, as done in the original TRAC intervention). These sessions are intended to last 30‐45 minutes.

A brief orientation session with the study coordinator was also added to enrollment procedures so that the interventionist did not need to spend time discussing the self-monitoring tasks or overall study procedures. This orientation session lasts between 15 and 20 minutes and includes a review of study protocols, training on the mobile breathalyzers and self-monitoring procedures, and scheduling future study tasks. Importantly, in response to concerns raised during the interviews, the study coordinator emphasizes the confidentiality of breathalyzer results and addresses any participant concerns about the breathalyzer during this session.

As an added measure to reduce study burden for this population, we reduced the number of required self-monitoring tasks; in the original TRAC intervention, participants completed both a breathalyzer reading and mobile survey twice each day (4 tasks total). Though completion rates for these were relatively high in the original intervention (median of 85% of mobile surveys and 78% of breathalyzers) [14], it was tested in an older adult population with fewer competing life demands than adolescents and young adults. In recognition of these and the concerns raised by participants, it was determined that for TRAC-adolescent and young adult, we would ask participants to complete one morning survey and one evening breathalyzer reading (2 tasks total), completed at the time of their choosing.

### Tailoring Intervention Content for Adolescent and Young Adult Survivors

**Figure 1.**
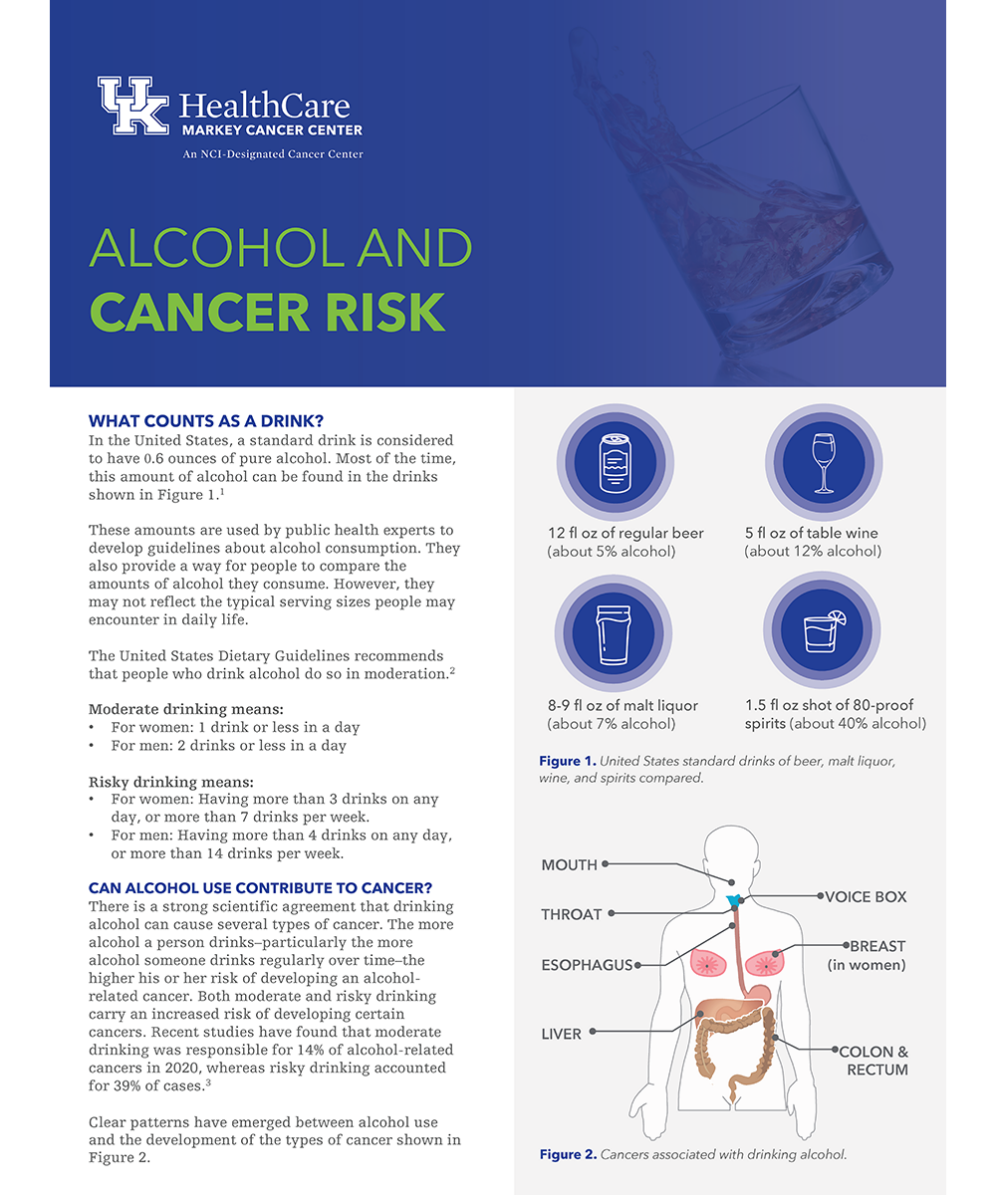
Excerpt from alcohol and cancer fact sheet.

For Session One, we developed a fact sheet that described the link between alcohol use and cancer, given that the topic is not often discussed in survivorship settings. This fact sheet (Can be found in Figure 1 for example) describes suggested drinking limits, the link between certain types of cancer and alcohol use, information about alcohol use and cancer recurrence and development of second primaries, how tobacco use and alcohol intersect to increase cancer risks, and other health effects of alcohol use. All participants receive this fact sheet in their participant manual, and the interventionist discusses it with them at the beginning of the intervention.

As mentioned, we also added a new session focused specifically on cancer-related triggers for alcohol use as reported through the key informant interviews (Session 3). We included content on fear of recurrence and surveillance, which addresses “scanxiety” and provides strategies for addressing cancer-related worry. These strategies include tips on how to find trustworthy information online, making a survivorship care plan, and suggestions for challenging negative thoughts, among others. Participants could choose between discussing financial toxicity or fertility concerns. For each topic, participants were encouraged to set actionable goals for addressing these concerns through seeking support or additional information, and they also received a resource guide to help them achieve those goals.

In addition, based on suggestions received from key informants to bolster adolescent and young adult engagement over the full study period, we added the option to track more health behaviors and outcomes through daily surveys (eg, mood, sleep quality, and physical activity). It was determined that participants would be allowed to track up to 3 of these additional health metrics and would receive a weekly report of their responses.

## Discussion

### Principal Findings

Both survivors and providers suggested that alcohol use is not routinely discussed in cancer care. This has been reported in previous research [23], though the literature in this area is scarce; in a large representative sample of US survivors, approximately 40% reported not discussing alcohol use with their clinician. Another study [24] of head and neck cancer survivors found that while only approximately 40% of survivors who drank recalled receiving advice from their oncology clinician regarding their alcohol use, those who received such advice were significantly more likely to reduce their alcohol use. These studies not only point to the need for developing approaches to address alcohol use in the context of cancer care, but also to the potential value of such approaches in reducing alcohol use among survivors.

Participants in the key informant interviews provided many suggestions for adapting TRAC content to better suit the adolescent and young adult survivor population. Several of these changes concerned reducing the time commitment of the intervention, including reducing the number of sessions from 8 to 4 and decreasing the number of required daily assessments. These changes were made to better accommodate the life circumstances of adolescents and young adults, many of whom are already struggling to cope with challenges relating to their education, career and employment, and intimate or familial relationships [25,26].

We also added cancer-specific triggers for alcohol use to the intervention to better tailor the content for adolescent and young adult survivors. Our goal in discussing cancer-specific triggers was to provide survivors with the necessary resources to manage the stress associated with these triggers and to increase coping self-efficacy, which in turn may help to reduce alcohol use as a coping mechanism [27,28]. Information relating to the financial concerns of survivors was added to the intervention. Financial toxicity, which can be described as the issues that arise in patients’ physical and mental health due to the costs of medical care, can have a significant impact on the lives of adolescent and young adult survivors—data show that adolescents and young adults see higher medical costs than their unaffected peers [29], and, depending on the type of cancer and treatments received, survivors may face challenges in entering or returning to work [30]. Previous research suggests that financial concerns and survivors’ mental health are closely related [31]. We also added content related to fertility and reproductive health concerns, which was discussed as a potential trigger by key informants and has been shown to be a common stressor for adolescent and young adult survivors. A qualitative study showed that survivors felt as though fertility was an afterthought in their cancer care and wanted more information about reproductive health [32]. Another study demonstrated the gaps in survivors’ knowledge about their own fertility and reproductive health [33], which, when left unaddressed, can be distressing for survivors. Finally, we also added content on cancer-related worry and fear of recurrence. These sources of stress have been found to have a negative impact on survivors’ overall quality of life [34], and survivors who report having a fear of recurrence or worry surrounding medical appointments or scans—“scanxiety”—report increased psychological distress [8,35]. Given the impact of these topics on adolescents’ and young adults’ mental health, it is not surprising that key informants suggested that they may also impact drinking.

In making the aforementioned changes to the intervention, the necessity of considering adolescents’ and young adults’ unique triggers for alcohol use and their life circumstances when developing interventions became clear, especially in light of the significant stressor of a cancer diagnosis, its treatment, and its physical and psychosocial sequelae. Obtaining feedback from members of this group and key stakeholders in their care was invaluable, and the willingness to participate and bolster care for this understudied group was a strong facilitator for this type of formative work. A barrier to adapting this intervention for adolescents and young adults was the diversity of members of the adolescent and young adult population, which spans all genders, includes people ranging from age 15 to 39 years who represent very different life stages, and includes people who were diagnosed as both adolescents and young adults. Thus, the population of adolescents and young adults is relatively heterogeneous, making it difficult to identify triggers that are relevant for the whole population. For that reason, we maintained core alcohol triggers (emotional, situational, and social) and added what are likely the most common cancer-specific triggers adolescents and young adults may experience (fear of recurrence, financial worry, and worry about fertility). To personalize the intervention and meet the need of maintaining brevity, adolescents and young adults are guided to choose their most salient core trigger and work through the content for fear of recurrence as a trigger, with the option of also completing financial worry or worry about fertility, depending on relevance to them. Future researchers aiming to adapt interventions for the adolescent and young adult population should take care to consider the heterogeneity of this group, obtain feedback from individuals representing various subgroups of this population, and consider offering opportunities for participants to tailor interventions to better suit their interests and circumstances.

### Limitations

This study is limited by its lack of racial or ethnic diversity among participants and its small sample size, though a large sample was not the purpose of this current study. Of the 15 key informants who participated in interviews, the majority were white, non-Hispanic. Though consistent with our cancer center’s catchment area, we acknowledge that this sample may not represent those of different racial or ethnic backgrounds. Our focus in this study was specifically on survivors who live in rural areas and may not represent survivors who live in urban or suburban areas. Further tailoring to the TRAC-adolescent and young adult intervention may be needed to adapt to the specific needs of populations not represented in the key informant interviews. In addition, current hazardous alcohol use was not a criterion for eligibility for survivors to participate; however, most did meet criteria for hazardous use.

### Conclusions

There is potential to increase alcohol intervention relevance and fit for adolescents and young adults by including tailored content relevant to their life experiences while also maintaining core components of such interventions, such as self-monitoring and goal-setting. Remote, brief interventions are important for ensuring acceptability. The new TRAC-adolescent and young adult intervention represents a potentially valuable tool in addressing high rates of hazardous alcohol use among this population and warrants further evaluation in randomized trials. As mentioned, this study was part of a larger project to develop and pilot a tailored alcohol reduction intervention for adolescent and young adult survivors of cancer. An open pilot trial and pilot randomized controlled trial (ClinicalTrials.gov NCT05087875) were completed to determine the feasibility and acceptability of this new intervention. Results of the RCT are forthcoming. If efficacious, TRAC-adolescent and young adult has the potential to provide an accessible, scalable intervention approach for addressing alcohol use among posttreatment adolescents and young adults.

## Supplementary material

10.2196/59949 Checklist 1 COREQ checklist

## Abbreviations

BrAC: Breath alcohol concentration
COREQ: Consolidated criteria for Reporting Qualitative research
HIPAA: Health Insurance Portability and Accountability Act
mHealth: mobile health
REDCap: Research Electronic Data Capture
TRAC: Tracking and Reducing Alcohol Consumption
